# Appetite loss during chemotherapy is associated with reduced dietary intake and quality of life in patients with gynecologic cancer: a prospective observational study

**DOI:** 10.1007/s00520-026-11276-8

**Published:** 2026-10-02

**Authors:** Ayami Koike, Rin Mizuno, Nozomi Higashiyama, Yoshihide Inayama, Maki Umemiya, Kaori Ikeda, Nozomi Kubo, Yu Tokushige, Takayuki Nagasawa, Noriomi Matsumura, Taito Miyamoto, Mana Taki, Koji Yamanoi, Ryusuke Murakami, Ken Yamaguchi, Masaki Mandai

**Affiliations:** 1 Department of Gynecology and Obstetrics, Graduate School of Medicine and Faculty of Medicine, Kyoto University, 54 Kawahara-cho, Shogoin, Sakyo-ku, Kyoto, 606-8507 Japan; 2 Department of Obstetrics and Gynecology, Shizuoka General Hospital, Shizuoka, Japan; 3 Department of Diabetes, Endocrinology and Nutrition, Kyoto University Graduate School of Medicine, Kyoto, Japan; 4 Department of Obstetrics and Gynecology, Kitano Hospital, Osaka, Japan; 5 Department of Obstetrics and Gynecology, Osaka Red Cross Hospital, Osaka, Japan; 6 Department of Obstetrics and Gynecology, Iwate Medical University School of Medicine, Iwate, Japan; 7 Department of Obstetrics and Gynecology, Kindai University Faculty of Medicine, Sakai, Japan; 8 Department of Obstetrics and Gynecology, Graduate School of Biomedical and Health Sciences, Hiroshima University, Hiroshima, Japan

**Keywords:** Gynecologic cancer, Chemotherapy, Appetite loss, Quality of life, Dietary intake, Taste change

## Abstract

**Purpose:**

Appetite loss is common during chemotherapy. However, its association with dietary intake and quality of life (QOL) in patients with gynecologic cancer remains unclear. This study aimed to clarify the dietary changes associated with appetite loss and investigate their association with QOL and adverse events.

**Methods:**

Patients with gynecologic cancer receiving chemotherapy were enrolled in this single-center longitudinal observational study. Dietary intake was assessed at baseline and before the third chemotherapy cycle using a diet history questionnaire. Appetite loss, QOL, and adverse events were evaluated using the EORTC QLQ-C30 and PRO-CTCAE. Sensitivity analyses addressed pre-existing appetite loss and postoperative effects. A complementary analysis of a separate cohort examined the association between appetite loss and longitudinal QOL changes.

**Results:**

Among 105 patients, 41 (39.0%) reported appetite loss before the third chemotherapy cycle. The appetite loss group showed greater reductions in energy and macronutrient intake, whereas macronutrient composition did not differ between the groups. Absolute micronutrient intake broadly decreased, but no difference remained significant after energy adjustment. Among adverse events, taste changes were most closely associated with appetite loss. Appetite loss was also associated with QOL decline, and dietary findings were directionally consistent in sensitivity analyses. The complementary analysis showed appetite loss was consistently associated with QOL fluctuations.

**Conclusion:**

Appetite loss during chemotherapy was associated with reduced dietary intake and lower QOL. Taste alterations were closely associated with appetite loss. These findings support appetite loss as a clinically relevant target for the supportive care of patients with gynecologic cancers.

**Supplementary Information:**

The online version contains supplementary material available at https://doi.org/10.1007/s00520-026-11276-8.

## Introduction

Despite recent advances in the treatment strategies for gynecologic cancers, cytotoxic chemotherapy remains the standard treatment option. Treatment-related adverse events can substantially impair patients' quality of life (QOL) [1], and have been associated with reduced physical functioning, treatment adherence, and poor prognosis [2]. Therefore, maintaining and improving the QOL during chemotherapy is an important clinical issue.

Nutritional impact symptoms that can cause appetite loss, such as nausea and taste alterations, are particularly common during chemotherapy [1, 3]. Appetite loss has been associated with reduced dietary intake [4, 5], impaired QOL, and adverse clinical outcomes [6, 7]. Notably, recent randomized controlled trials have suggested that improving appetite may lead to better QOL, reduce severe adverse events, and improve the maintenance of chemotherapy dose intensity [8].

However, most previous studies have focused on specific populations, such as patients with advanced cancer or those with gastrointestinal or lung cancers [8, 9]. In contrast, patients with gynecologic cancers often receive chemotherapy in an adjuvant setting, including those with relatively early-stage disease. Furthermore, unlike gastrointestinal or head and neck cancers, which directly impair oral intake, swallowing, or digestion, gynecologic malignancies generally do not directly interfere with these functions. The clinical significance of appetite loss in this population has not been sufficiently investigated. In particular, the specific dietary changes associated with appetite loss and their clinical implications remain poorly understood.

Therefore, this study aimed to investigate the clinical significance of appetite loss during chemotherapy in patients with gynecologic cancers. The primary objective of this study was to characterize the dietary changes associated with appetite loss, including changes in food groups and nutrient intake. The secondary objectives were to evaluate the association between appetite loss and other adverse events and to examine its relationship with QOL. To further evaluate the clinical relevance of appetite loss, a complementary analysis was conducted using a separate multicenter cohort to examine whether appetite loss was associated with longitudinal changes in QOL during treatment.

## Methods

### Study design

This study consisted of analyses of two patient cohorts. The primary analysis was performed using a prospective single-center cohort with a detailed dietary assessment and included both primary and secondary outcomes. A complementary analysis was conducted using a separate multicenter cohort with longitudinal patient-reported outcome (PRO) data to provide additional evidence on the relationship between appetite loss and QOL.

### Participants

#### Primary analysis cohort

Patients newly diagnosed with gynecologic malignancies were prospectively recruited from Kyoto University Hospital between July 2022 and March 2026. Eligible participants met the following criteria: (ⅰ) histologically confirmed diagnosis of cancer and scheduled to receive first-line chemotherapy, (ⅱ) age ≥ 18 years, and (ⅲ) ability to read and write Japanese. The exclusion criteria were as follows: (ⅰ) diagnosis of recurrent disease, (ⅱ) receiving concurrent radiotherapy, and (ⅲ) the presence of another active malignancy. Dietary intake and QOL were assessed at two time points: at baseline, before the initiation of treatment (1–2 days before surgery or one day before the first chemotherapy cycle), and at follow-up, one day before the third chemotherapy cycle. Patients who did not complete the baseline or follow-up dietary questionnaires were excluded.

#### Complementary analysis cohort

A multicenter prospective cohort of 127 patients with gynecologic malignancies receiving anticancer treatment was enrolled at five institutions in Japan between June 2020 and March 2024. The QOL and treatment-related symptoms were longitudinally assessed using PRO measures over a 15-week observation period. Additional details are provided in the Supplementary Methods.

These studies were approved by the Ethics Committee of the Graduate School and Faculty of Medicine at Kyoto University (approval number: C1509), and all participants provided written informed consent.

### Data collection

#### Dietary assessment

Dietary intake information was assessed using a brief validated self-administered diet history questionnaire (BDHQ) [10, 11]. The questionnaire assessed dietary habits during the preceding month, and daily energy intake, nutrient intake, and food group consumption were calculated from more than 50 food and beverage items using the validated BDHQ algorithm. Patients were excluded if they reported significantly abnormal energy intake (< 500 or > 3500 kcal/day).

#### QOL and appetite loss

QOL and symptoms, including appetite loss, were assessed using the European Organization for Research and Treatment of Cancer Quality of Life Questionnaire Core 30 (EORTC QLQ-C30) [12]. The questionnaire included global health status (GHS)/QOL, five functional scales, and nine symptom scales/items. Higher scores on the GHS/QOL and functional scales indicated better QOL, whereas higher symptom scores indicated greater symptom severity. Appetite loss was assessed using item 13 (“Have you lacked appetite?”), scored on a four-point Likert scale (1 = not at all, 2 = a little, 3 = quite a bit, and 4 = very much), and linearly transformed to a standardized 0–100 scale according to the EORTC scoring manual [13]. Based on appetite loss prior to the third chemotherapy cycle (reflecting the preceding week), patients were classified into two groups: no appetite loss (score = 0, corresponding to "not at all") and appetite loss (score > 0, corresponding to "a little" or higher).

This classification reflects appetite loss status at a single time point and does not distinguish pre-existing from newly developed appetite loss (see Sensitivity Analyses). This cutoff was used to capture the presence of appetite loss, including mild symptoms, because the study aimed to examine whether patient-reported appetite loss, even when mild, was associated with dietary and QOL changes, rather than to identify clinically severe appetite loss.

#### Adverse events

Chemotherapy-related adverse events were assessed before the third chemotherapy cycle using the Japanese version of the PRO version of the Common Terminology Criteria for Adverse Events (PRO-CTCAE) severity domain [14]. Seven symptoms associated with appetite loss and dietary intake (dry mouth, mouth sores, taste changes, nausea, constipation, diarrhea, and fatigue) were selected based on their clinical relevance. Decreased appetite was also assessed using the PRO-CTCAE severity domain. Symptom severity was rated on a five-point Likert scale (0 = none to 4 = very severe), with higher scores indicating greater severity.

#### Demographic and clinical information

At baseline, a standard self-administered questionnaire was used to collect socio-demographic and lifestyle information, including living arrangements and income. Weight loss was assessed by comparing the baseline body weight with the body weight at the last available chemotherapy cycle (generally around the sixth cycle), which was considered less affected by postoperative weight changes and more reflective of dietary intake during chemotherapy. Clinicopathological data, laboratory test results, and information on nutritional counseling were obtained from the electronic medical records. Nutritional counseling was defined as cancer-related dietary counseling provided between treatment initiation and the assessment before the third chemotherapy cycle. Serum albumin levels and total lymphocyte counts were measured at baseline and before the third chemotherapy cycle. The Prognostic Nutritional Index (PNI) was calculated as an indicator of nutritional status using the following formula: PNI = (10 × serum albumin [g/dL]) + (0.005 × total lymphocyte count [/mm^3^]) [15].

### Study outcomes

The primary outcome was the change in dietary intake between baseline and chemotherapy according to appetite loss status, including changes in food groups and nutrient intake. The secondary outcomes were the associations of appetite loss with other adverse events and QOL.

### Statistical analysis

Baseline clinicopathological characteristics were compared between the groups using the Mann–Whitney *U* test for continuous variables and the chi-square test or Fisher's exact test for categorical variables, as appropriate. Continuous variables are presented as medians and interquartile ranges (IQR), and categorical variables are presented as numbers and percentages.

For the primary analysis, changes in dietary intake were calculated as the difference between baseline and during chemotherapy. Between-group differences were assessed using the Mann–Whitney *U* test. For macronutrient and food group intake, effect sizes were defined as the difference in median changes between groups, with 95% confidence intervals (CIs) estimated using a nonparametric bootstrap procedure with 3,000 resamples and sampling with replacement. Analyses of micronutrient intake focused on 16 clinically relevant micronutrients commonly used in nutritional assessments. Energy-adjusted intake was also evaluated using nutrient density (micronutrient intake per 1000 kcal of energy intake). Effect sizes were quantified using Hedges' *g* to facilitate interpretation of the magnitude of between-group differences, and *p* values were adjusted for multiple comparisons using the Benjamini–Hochberg false discovery rate (FDR) procedure.

Associations between appetite loss and adverse events were evaluated using multiple linear regression models, with appetite loss scores as the dependent variable and PRO-CTCAE symptom scores as explanatory variables, adjusted for age and body mass index (BMI).

Spearman’s rank correlation analysis was used to examine the associations between appetite loss scores and QOL measures, including the GHS/QOL and the five functional scales of the EORTC QLQ-C30. Spearman’s rank correlation was also used to assess the association between the EORTC QLQ-C30 appetite loss item (item 13) and PRO-CTCAE decreased appetite severity among patients with available PRO-CTCAE data.

For the complementary analysis, machine learning models were developed to evaluate the association between symptom changes and longitudinal changes in QOL. QOL change was defined as a ≥ 10-point change in the EORTC QLQ-C30 GHS/QOL score, corresponding to the minimal important change threshold [16]. The model interpretation was performed using Shapley’s additive explanations. Further methodological details are provided in the Supplementary Methods.

### Sensitivity analyses

Sensitivity analyses were conducted to evaluate the robustness of the primary findings. First, separate analyses of covariance (ANCOVA) models were used to examine the association between appetite loss before the third cycle and energy and macronutrient intakes in the third cycle, with each model adjusted for the corresponding baseline intake, baseline appetite loss score, and age. To account for the potential influence of postoperative factors, these analyses were repeated after restricting the analysis to patients who received postoperative adjuvant chemotherapy, followed by further adjustment for surgical approach (open vs minimally invasive surgery) and the interval from surgery to chemotherapy initiation. Second, to minimize the potential influence of pre-existing appetite loss, analyses were repeated among patients who reported no appetite loss at baseline. Changes in energy and macronutrient intakes from baseline to the third chemotherapy cycle were compared between patients who developed appetite loss by the third cycle and those who remained free of appetite loss using the Mann–Whitney *U* test. Third, patients were additionally categorized into three groups according to appetite loss severity (“not at all,” “a little,” and “quite a bit” or “very much”). Trends in energy and macronutrient intake were evaluated using Spearman’s rank correlation coefficients.

All statistical analyses were performed using Python version 3.12.12 (Python Software Foundation, Wilmington, DE, USA). *p* values < 0.05 were considered statistically significant.

## Results

### Patient characteristics

The study enrollment flowchart is presented in Supplementary Fig. 1. A total of 153 patients were recruited and completed the baseline dietary questionnaire. Of these, 48 were excluded because of withdrawal, discontinuation of chemotherapy, treatment at another hospital, incomplete questionnaire data, or loss to follow-up, leaving 105 patients for analysis. Appetite loss before the third chemotherapy cycle was reported by 41 (39.0%) patients. The distribution of each symptom scale during chemotherapy is shown in Supplementary Table 1. The EORTC QLQ-C30 appetite loss item was positively correlated with PRO-CTCAE decreased appetite severity (*n* = 81; Spearman’s *ρ* = 0.71, *p* < 0.001), with the distribution according to appetite loss status presented in Supplementary Table 2.

Table 1 presents baseline clinical characteristics stratified by appetite loss status. Among patients who received postoperative adjuvant chemotherapy, the interval from surgery to chemotherapy initiation was significantly shorter in the appetite loss group than in the no appetite loss group (34.0 [28.0 to 40.5] vs. 40.0 [32.0 to 47.5] days, median [IQR]; *p* = 0.03). No other statistically significant differences in baseline characteristics were observed between the appetite loss and no appetite loss groups. Patients in the appetite loss group tended to be younger (53.0 [46.0 to 64.0] vs. 58.0 [53.8 to 66.0] years; *p* = 0.06) and more frequently underwent open surgery (77.1% vs. 61.8%). Ovarian cancer was more common in the appetite loss group (46.3%), whereas endometrial cancer was the most common cancer type in the no appetite loss group (48.4%). Baseline appetite loss scores were also numerically higher in the appetite loss group, although the difference was not statistically significant (33.3 [0 to 33.3] vs. 0 [0 to 33.3], median [IQR]; *p* = 0.18).

**Table 1:** Baseline clinicopathologic characteristics of the patients by appetite loss status during chemotherapy

Characteristics	Appetite loss (*n* = 41) *n* (%) or median [IQR]	No appetite loss (*n* = 64) *n* (%) or median [IQR]	*p* value
Age (years)	53.0 [46.0 to 64.0]	58.0 [53.8 to 66.0]	0.06
BMI (kg/m^2^)	21.9 [19.5 to 27.7]	21.0 [19.7 to 24.0]	0.29
Cancer type			
Ovary	19 (46.3)	24 (37.5)	0.49
Endometrium	14 (34.1)	31 (48.4)	
Cervix	7 (17.1)	7 (10.9)	
Others	1 (2.4)	2 (3.1)	
Cancer stage			
Ⅲ/Ⅳ, *n* (%)	16 (39.0)	24 (37.5)	1.0
Chemotherapy regimen			
Taxanes ± platinum, *n* (%)	35 (85.4)	59 (92.2)	0.33
Type of chemotherapy			
Neoadjuvant	6 (14.6)	9 (14.1)	1.0
Adjuvant	35 (85.4)	55 (85.9)	
Surgical approach^a^			
Open	27 (77.1)	34 (61.8)	0.17
Minimally invasive^b^	8 (22.9)	21 (38.2)	
Surgery-to-chemotherapy interval (days)^a^	34.0 [28.0 to 40.5]	40.0 [32.0 to 47.5]	0.03
Living alone^c^	5 (13.2)	13 (21.3)	0.42
Household income (*10^6^JPY)			
< 5	16 (39.0)	28 (43.8)	0.68
5–10	15 (36.6)	21 (32.8)	
> 10	3 (7.3)	8 (12.5)	
No reply	7 (17.1)	7 (10.9)	
Baseline appetite loss score	33.3 [0 to 33.3]	0 [0 to 33.3]	0.18
Serum albumin (g/dL)	4.0 [3.5 to 4.2]	4.1 [3.7 to 4.3]	0.51
PNI	41.1 [35.9 to 43.1]	41.7 [37.5 to 43.8]	0.58

*JPY* Japanese Yen, *PNI* Prognostic Nutritional Index (Onodera’s PNI)

^a^Among patients who underwent surgery before chemotherapy (*n* = 35 in the appetite loss group and *n* = 55 in the no appetite loss group)

^b^Minimally invasive surgery included laparoscopic and robotic approaches

^c^Percentages calculated among patients with known data

### Energy and macronutrient intake

**Fig. 1:**
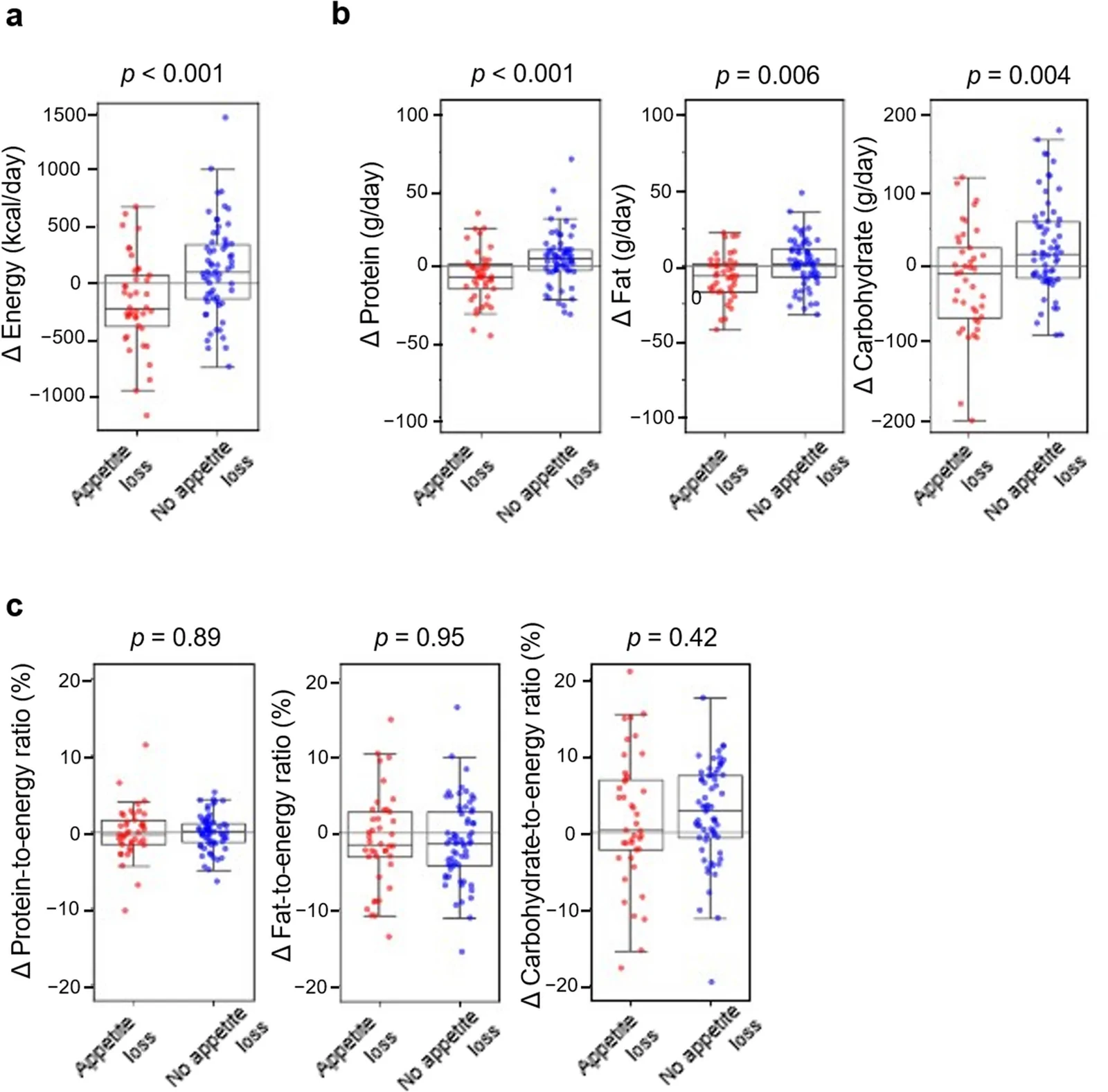
Changes in daily dietary intake from baseline to the third cycle of chemotherapy stratified by appetite loss status. (**a**) energy intake, (**b**) macronutrient intake (protein, fat, carbohydrate), and (**c**) PFC ratio (% of total energy from protein, fat, carbohydrate). Box-and-scatter plots indicate medians, interquartile ranges, and individual values. *p* values were calculated using the Mann–Whitney *U* test

Figure 1 shows a comparison of changes in daily energy and macronutrient intake between the two groups. Energy intake decreased to a greater extent in the appetite loss group (median difference − 320.8 kcal/day; 95% CI, − 454.5 to − 119.3; *p* < 0.001; Fig. 1a). The same pattern was observed for protein (− 12.0 g/day; 95% CI, − 16.7 to − 4.9; *p* < 0.001), fat (− 8.3 g/day; 95% CI, − 15.2 to − 1.7; *p* = 0.006), and carbohydrate (− 25.6 g/day; 95% CI, − 67.1 to − 2.7; *p* = 0.004) intake (Fig. 1b). Group medians and IQRs are given in Supplementary Table 3. In contrast, no clear differences were observed between the two groups in changes in the PFC ratio (% of total energy from proteins, fats, and carbohydrates) (Fig. 1c).

Sensitivity analyses using ANCOVA showed that appetite loss before the third cycle was associated with lower energy, protein, fat, and carbohydrate intakes after adjustment for the corresponding baseline intake, baseline appetite loss score, and age. In the postoperative cohort, the associations remained significant for energy, protein, and carbohydrate intake, but not for fat intake, and these findings were unchanged after further adjustment for surgical approach and the interval from surgery to chemotherapy initiation (Supplementary Table 4). In a sensitivity analysis restricted to 49 patients without appetite loss at baseline, 16 (32.7%) developed incident appetite loss by the third chemotherapy cycle. Changes in energy, protein, fat, and carbohydrate intake were consistently more negative among patients with incident appetite loss than among those who remained free of appetite loss, although the between-group difference was statistically significant only for carbohydrate intake (*p* = 0.040; Supplementary Table 5). In an additional sensitivity analysis, greater severity of appetite loss was associated with larger reductions in energy and macronutrient intake (Supplementary Table 6).

### Food intake

**Fig. 2:**
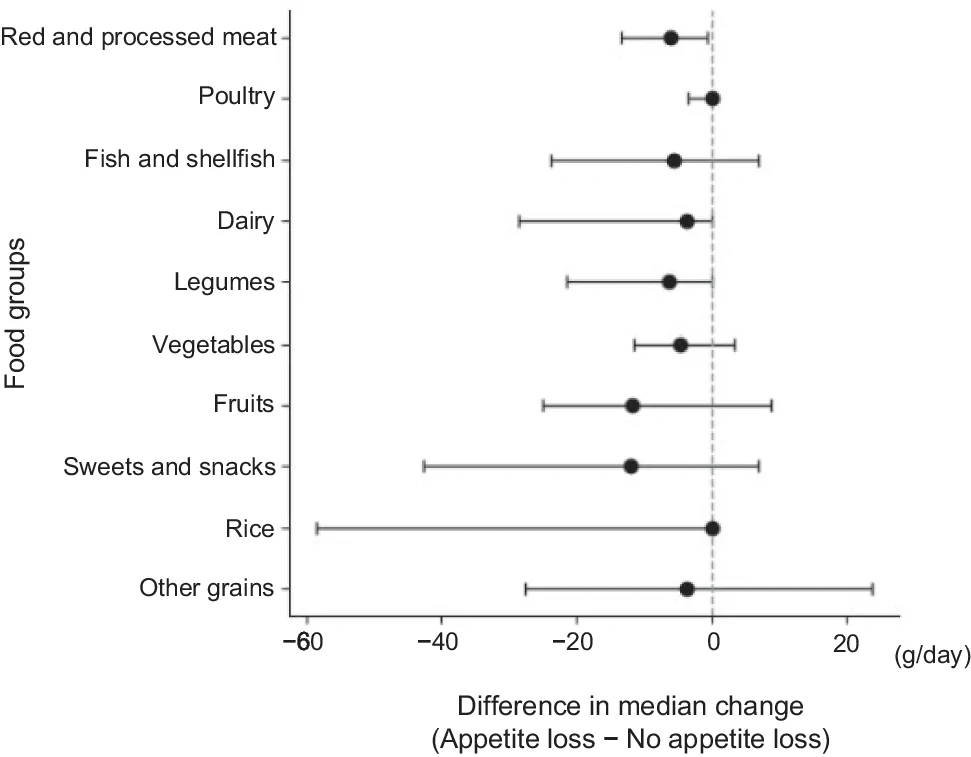
Differences in median changes in food group intake between patients with and without appetite loss during chemotherapy. Points represent the median differences (Appetite loss group − No appetite loss group, g/day), and horizontal lines indicate 95% confidence intervals. Negative values indicate greater reductions in the appetite loss group. Other grains include bread, pasta, *udon*, *soba*, and *ramen* (Japanese noodles)

Figure 2 compares the changes in the daily intake of food groups between the two groups. Compared with the no appetite loss group, the appetite loss group showed a greater decrease in red and processed meat intake, with a median difference of − 6.1 g/day (95% CI, − 13.4 to − 0.6). Dairy products and legumes also showed numerically greater decreases in the appetite loss group, with a median difference of − 3.8 g/day (95% CI, − 28.7 to 0.0) and − 6.2 g/day (95% CI, − 21.5 to 0.0), respectively. No differences were observed between the two groups in poultry, fish and shellfish, vegetables, fruits, sweets and snacks, or carbohydrate sources (e.g., rice and other grains).

### Micronutrient intake

**Fig. 3:**
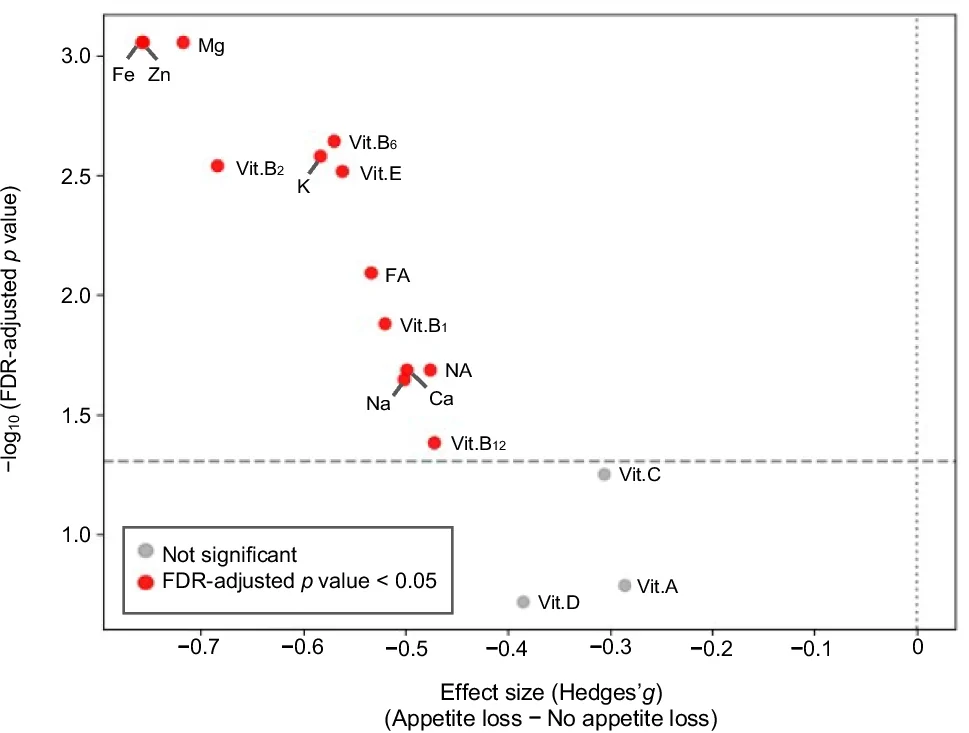
Effect sizes of changes in daily micronutrient intake between patients with and without appetite loss during chemotherapy. The x-axis indicates effect sizes calculated as Hedges’ *g* (Appetite loss group − No appetite loss group). The y-axis represents − log_10_(FDR-adjusted *p* values). Red points indicate nutrients with FDR-adjusted *p* values < 0.05. The horizontal dashed line indicates the significance threshold corresponding to an FDR-adjusted *p* value of 0.05 (− log_10_[FDR-adjusted *p* value] = 1.3). A tabular summary of effect sizes and FDR-adjusted *p* values is provided in Supplementary Table 7. Fe, iron; Zn, zinc; Mg, magnesium; K, Potassium; Na, Sodium; Ca, Calcium; NA, Niacin; FA, Folate; Vit.B_1_, Vitamin B_1_; Vit.B_2_, Vitamin B_2_; Vit.B_6_, Vitamin B_6_; Vit.B_12_, Vitamin B_12_; Vit.A, Vitamin A; Vit.C, Vitamin C; Vit.D, Vitamin D; Vit.E, Vitamin E

Figure 3 and Supplementary Table 7 show the effect sizes of the changes in micronutrient intake between patients with and without appetite loss. Of the 16 micronutrients examined, 13 showed significantly larger decreases in the appetite loss group after FDR correction (FDR-adjusted *p* < 0.05): iron, zinc, magnesium, and vitamin B₂ had the largest effect sizes (Hedges’ *g* =  − 0.68 to − 0.76), and the remaining nine were small to moderate (− 0.47 to − 0.58). The other three micronutrients did not differ significantly. However, after adjusting for energy intake using nutrient density (micronutrient intake per 1000 kcal), none of the between-group differences was statistically significant.

### Adverse events associated with appetite loss

To explore the symptom patterns associated with appetite loss during chemotherapy, multivariable linear regression analysis was conducted (Table 2). Taste changes were significantly associated with the appetite loss scale (*β* coefficient, 8.7 (95% CI, 1.3 to 16.1); *p* = 0.02), whereas none of the other adverse events, including fatigue, constipation, nausea, mouth sores, diarrhea, and dry mouth, showed significant associations with the appetite loss scale. Among the covariates, neither age nor BMI was significantly associated with the outcome.

**Table 2:** Factors associated with appetite loss during chemotherapy by multiple linear regressions

Variable	*β* coefficient	(95% CI)	Standardized *β*	*p* value
**Adverse events**				
Taste changes	8.7	(1.3 to 16.1)	0.35	0.02
Fatigue	5.2	(− 2.4 to 12.8)	0.21	0.18
Constipation	− 3.4	(− 9.2 to 2.4)	− 0.14	0.25
Nausea	3.2	(− 6.4 to 12.8)	0.12	0.51
Mouth sores	− 4.0	(− 12.9 to 4.8)	− 0.11	0.36
Diarrhea	1.0	(− 6.8 to 8.7)	0.03	0.80
Dry mouth	− 0.9	(− 8.7 to 7.0)	− 0.03	0.83
**Covariates**				
Age	− 0.2	(− 0.7 to 0.4)	− 0.07	0.53
BMI	1.0	(− 0.2 to 2.1)	0.19	0.10

### Association between changes in appetite loss, QOL, and nutritional indicators

Changes in appetite loss scale scores were negatively correlated with changes in GHS/QOL scores (Spearman’s *ρ* =  − 0.31, *p* = 0.002; Table 3). Among the functional scales, stronger negative correlation coefficients were observed for role functioning (*ρ* =  − 0.49, *p* < 0.001), cognitive functioning (*ρ* =  − 0.42, *p* < 0.001), social functioning (*ρ* =  − 0.35, *p* < 0.001), and emotional functioning (*ρ* =  − 0.28, *p* = 0.006) than for physical functioning (*ρ* =  − 0.16, *p* = 0.11). Among the symptom scales, changes in appetite loss were positively correlated with changes in almost all other symptom scales.

**Table 3:** Correlations between changes in appetite loss scale and changes in EORTC QLQ-C30 scores

Variable	*n*	Spearman’s *ρ*	*p* value
**Global health status/QOL, functional scales**			
∆GHS/QOL	96	− 0.31	0.002
∆Physical functioning	98	− 0.16	0.11
∆Role functioning	97	− 0.49	< 0.001
∆Emotional functioning	96	− 0.28	0.006
∆Cognitive functioning	97	− 0.42	< 0.001
∆Social functioning	96	− 0.35	< 0.001
**Symptom scales**			
∆Fatigue scale	97	0.43	< 0.001
∆Nausea scale	97	0.50	< 0.001
∆Pain scale	96	0.38	< 0.001
∆Dyspnea scale	99	0.29	0.003
∆Insomnia scale	100	0.42	< 0.001
∆Constipation scale	97	0.27	0.008
∆Diarrhea scale	94	0.30	0.004
∆Financial difficulties scale	95	0.13	0.20

In contrast, no significant association was observed between appetite loss and the objective nutritional indicators. The proportion of patients experiencing > 5% weight loss was similar between the groups (26.8% vs. 21.9%, *p* = 0.56), and no significant differences were found in the changes in serum albumin levels or PNI (Supplementary Table 8). Additionally, nutritional counseling was provided to 8 patients (7.6%), with no significant difference between the appetite loss and no appetite loss groups (12.2% vs. 4.7%, *p* = 0.26).

### Complementary analysis of the association between appetite loss and changes in QOL

The patient characteristics and score distributions of the complementary cohort are presented in Supplementary Tables 9 and 10. The GHS/QOL scores showed within-cycle fluctuations, decreasing at week 1 and improving at week 2 (Supplementary Fig. 2a). Across the machine learning models, appetite loss consistently emerged as one of the most influential features associated with both QOL deterioration and improvement (Supplementary Fig. 2b and 2c). The detailed model performance metrics are provided in Supplementary Fig. 3 and Supplementary Table 11.

## Discussion

In this prospective cohort study of patients with gynecologic malignancies undergoing chemotherapy, appetite loss was associated with generalized reductions in dietary intake, accompanied by a broad decrease in micronutrient intake, which largely paralleled the reduction in overall energy intake. In contrast, macronutrient composition (PFC ratio) remained unchanged. The sensitivity analysis restricted to patients without appetite loss at baseline showed directionally consistent changes in dietary intake, although the small sample size warrants cautious interpretation.

Previous studies have reported that patients with appetite loss demonstrate lower energy and protein intakes and greater weight loss [4]. In contrast, we found no significant association between appetite loss and body weight, serum albumin level, or PNI. This discrepancy may reflect differences between studies in patient characteristics, such as cancer type, treatment regimen, and disease stage, as well as observation periods and dietary assessment methods.

Although the macronutrient composition remained unchanged, appetite loss was associated with changes at the food group level, potentially reflecting altered food preferences during chemotherapy. In particular, the intake of red and processed meats tended to decrease in the appetite loss group. This finding is consistent with previous reports showing that meat is the most commonly aversive food during anticancer treatment [17] and that impaired taste function is associated with reduced preference for protein-rich foods [18].

Although appetite loss was associated with an absolute decrease in a broad range of micronutrients, none of the between-group differences remained significant after energy adjustment, suggesting that micronutrient density was largely preserved. However, patients with severe or prolonged energy restriction may fail to meet their absolute micronutrient requirements. Among the micronutrients examined, zinc is of particular interest because of its established role in taste function [19, 20]. Experimental studies have suggested that even mild zinc deficiency can impair taste function [21]. One possible explanation is that lower overall food intake associated with appetite loss may be accompanied by reduced zinc intake, which may in turn be associated with taste alterations. Future studies incorporating biochemical assessments of zinc status are warranted to clarify this potential mechanism.

In this study, appetite loss was consistently associated with a decline in QOL. Our complementary analysis identified appetite loss as one of the most influential features associated with QOL fluctuations during the chemotherapy cycle, with QOL declining in the first week after treatment and recovering thereafter. These findings suggest that clinically relevant QOL changes may occur between hospital visits when patients are less closely monitored by healthcare providers, and that appetite loss may be associated with these temporal changes.

Correlations with appetite loss were stronger for role, cognitive, social, and emotional functioning than for physical functioning. Consistent with this finding, previous studies have linked poor food intake with depressive symptoms and reduced QOL. Supporting this relationship, psychotropic medications such as olanzapine and mirtazapine have been reported to improve both appetite and QOL in patients with cancer [8, 22]. These observations suggest that appetite loss may be more closely related to psychological and social well-being than physical functioning.

This study has several limitations. First, dietary intake was assessed using a food frequency questionnaire, which is subject to systematic errors such as reporting bias. However, we used a validated Japan-specific dietary assessment tool [10, 11], and focused on within-individual changes, which may have reduced measurement error. In addition, appetite loss was assessed over the preceding week, whereas dietary intake reflected the preceding month; therefore, these measures did not cover the same time frame, and the temporal direction or causality of their association cannot be determined. The findings should therefore be interpreted as an association between appetite loss and changes in dietary intake rather than as a sequential or causal relationship. Furthermore, because our definition of appetite loss included mild symptoms, the findings should be interpreted in the context of predominantly mild appetite loss.

Second, a selection bias cannot be excluded, as patients with more severe appetite loss or poorer nutritional status may have been less likely to participate or complete the study. Consequently, these findings may reflect patients with relatively stable conditions. Although nutritional counseling was provided to only a small proportion of patients and did not significantly differ by appetite loss status, its potential influence on dietary intake and QOL cannot be completely excluded. In addition, although the observed associations remained significant for energy, protein, and carbohydrate intake after adjustment for surgical approach and the surgery-to-chemotherapy interval, residual confounding by postoperative recovery and unmeasured surgery-related symptoms cannot be fully excluded, given that most patients received postoperative adjuvant chemotherapy. Furthermore, given the moderate sample size and the potentially unmeasured confounding factors associated with appetite loss, these findings do not allow for definitive conclusions.

Third, because biochemical parameters such as serum zinc concentration were not measured, it remains unclear whether the observed reduction in dietary zinc intake reflects an actual physiological deficiency. Therefore, the proposed association between reduced zinc intake and taste alterations remains hypothetical.

Fourth, although EORTC QLQ-C30 appetite loss was correlated with PRO-CTCAE decreased appetite severity, its correspondence with CTCAE grading remains unclear. In addition, appetite loss and taste alterations were not assessed after completion of chemotherapy; therefore, whether these symptoms recovered following treatment completion remains unknown.

Despite these limitations, this study has several important clinical implications. Because appetite loss and taste alterations are common during chemotherapy, early recognition and nutritional interventions are essential. The recent introduction of electronic PRO (ePRO) may support the monitoring of changes outside hospital visits [23]. Management should include dietary counselling [24, 25], tailored modifications to accommodate taste changes, and, when necessary, oral nutritional supplements. Such interventions may help support adequate dietary intake and QOL during chemotherapy. Further longitudinal studies incorporating biochemical assessment of micronutrients and post-treatment follow-up are needed to clarify the relationships between appetite, nutrition, taste alterations, and QOL.

## Conclusion

In this prospective study of patients with gynecologic cancers undergoing chemotherapy, appetite loss was associated with overall reductions in dietary intake, accompanied by decreases in absolute micronutrient intake, which were largely explained by reduced energy intake without substantial changes in macronutrient composition. Appetite loss was also associated with impaired QOL, while taste alterations were closely associated with appetite loss. The complementary analysis further suggested that appetite loss may be associated with within-cycle QOL fluctuations during treatment. These findings highlight appetite loss as a clinically relevant symptom and a potential target for supportive care during chemotherapy in gynecologic oncology.

## Supplementary Information

Below is the link to the electronic supplementary material.

Supplementary file1 (PDF 540 KB)

## Data Availability

The datasets used and analyzed in this study are available upon reasonable request.
