# Supplementary material for: Appetite loss during chemotherapy is associated with reduced dietary intake and quality of life in patients with gynecologic cancer: a prospective observational study
Source: Support Care Cancer. 2026 Oct 2;34(10):1046. doi: 10.1007/s00520-026-11276-8 (PMC13633162; doi:10.1007/s00520-026-11276-8)
Supplement: Supplementary file 1 [file 520_2026_11276_MOESM1_ESM.pdf]

Title: Appetite Loss During Chemotherapy Is Associated with Reduced Dietary Intake and Quality of Life in Patients with Gynecologic Cancer: A Prospective Observational Study

journal name: Supportive Care in Cancer

Authors: Ayami Koike, M.D.[1], Rin Mizuno, M.D., Ph.D.[1], Nozomi Higashiyama, M.D.[1], Yoshihide Inayama, M.D., Ph.D.[1], Maki Umemiya, M.D.[1,] Kaori Ikeda, M.D., Ph.D.[2], Nozomi Kubo, M.D.[1,3], Yu Tokushige, M.D.[4], Takayuki Nagasawa, M.D.[5], Noriomi Matsumura, M.D., Ph.D.[6], Taito Miyamoto, M.D., Ph.D.[1], Mana Taki, M.D., Ph.D.[1], Koji Yamanoi, M.D., Ph.D.[1], Ryusuke Murakami, M.D., Ph.D.[1], Ken Yamaguchi, M.D., Ph.D.[1,7], Masaki Mandai, M.D., Ph.D.[1]

Affiliations:

[1] Department of Gynecology and Obstetrics, Graduate School of Medicine and Faculty of Medicine, Kyoto University, Kyoto, Japan

[2] Department of Diabetes, Endocrinology and Nutrition, Graduate School of Medicine, Kyoto University, Kyoto, Japan

[3] Department of Obstetrics and Gynecology, Kitano Hospital, Osaka, Japan

[4] Department of Obstetrics and Gynecology, Osaka Red Cross Hospital, Osaka, Japan

[5] Department of Obstetrics and Gynecology, Iwate Medical University School of Medicine, Iwate, Japan

[6] Department of Obstetrics and Gynecology, Kindai University Faculty of Medicine, Sakai, Japan

[7] Department of Obstetrics and Gynecology, Graduate School of Biomedical and Health Sciences, Hiroshima University, Hiroshima, Japan

Corresponding Author: Rin Mizuno, MD, PhD

Department of Gynecology and Obstetrics, Graduate School of Medicine and Faculty of Medicine, Kyoto University

54 Kawahara-cho, Shogoin, Sakyo-ku, Kyoto 606-8507, Japan

Tel: 81-75-751-3269

Fax: 81-75-761-3967

Email: rin0617@kuhp.kyoto-u.ac.jp

Supplementary Methods for Complementary analysis

Study design and participants

This multicenter prospective observational study was conducted at Kyoto University Hospital, Iwate Medical University Hospital, Kitano Hospital, Kindai University Hospital, and Osaka Red Cross Hospital. Inclusion criteria were as follows: (i) confirmed diagnosis of gynecologic cancer, (ii) currently undergoing chemotherapy, radiotherapy, or molecular targeted therapy, and (iii) the ability to download and operate a mobile application on their personal smartphone. The exclusion criterion was the inability to operate the mobile application on a smartphone.

Data collection details

Participants installed a designated mobile application on their personal smartphones and received instructions from the research team. Using this application, electronic patient-reported outcomes (ePROs) were collected longitudinally over a 15-week observation period.

The European Organization for Research and Treatment of Cancer Quality of Life Questionnaire Core 30 (EORTC QLQ-C30) was used to assess overall QOL and treatment-related symptoms, including the global health status (GHS)/QOL score and symptom scales (fatigue, nausea, pain, dyspnea, insomnia, appetite loss, constipation, and diarrhea), while the Patient-Reported Outcomes version of the Common Terminology Criteria for Adverse Events (PRO-CTCAE) severity domain was used to assess numbness, a common symptom during chemotherapy for gynecologic malignancies, and the total score of the Patient Health Questionnaire-9 (PHQ-9) [1] was used to assess depressive symptoms. The EORTC QLQ-C30 and PRO-CTCAE were administered weekly, whereas the PHQ-9 was administered every two weeks, in accordance with their respective recall periods.

Data analysis details

Classification modeling was performed at the patient-timepoint level. Two separate binary classification models were developed to predict QOL deterioration and QOL improvement. Deterioration was defined as a decrease of ≥10 points in GHS/QOL scores from the previous assessment, and improvement was defined as an increase of ≥10

points. For each symptom, a within-patient change feature was calculated as the difference between the symptom score at each time point and the patient-specific moving median over the preceding 28 days, excluding the current day. These change features were used as explanatory variables.

The algorithms used to develop the models were random forest (RF), Light Gradient Boosting Machine (LightGBM), and eXtreme Gradient Boosting (XGBoost). Data were split at the patient level into training and test sets to ensure that observations from the same individual were not distributed across different datasets. Hyperparameter tuning for each model was performed within the training data using Group 5-Fold cross-validation with patient ID as the grouping variable. Grid Search Cross Validation (CV) was used for RF and XGBoost, while Randomized Search CV was applied for LightGBM. A fixed random seed was used to ensure reproducibility. We assessed the performance of QOL classification models based on F1-score, accuracy, sensitivity, specificity, positive predictive value, negative predictive value and area under the curve (AUC). Finally, the explanatory power of the classification model was analyzed, using Shapley additive explanations (SHAP). SHAP analysis was performed using the random forest model, which demonstrated the most balanced discriminative performance across evaluation metrics. Data were analyzed using Python 3.12.12.

Figure S1. Flow diagram of patient selection for the data analyses

FFQ food frequency questionnaire, *CCRT* concurrent chemoradiotherapy

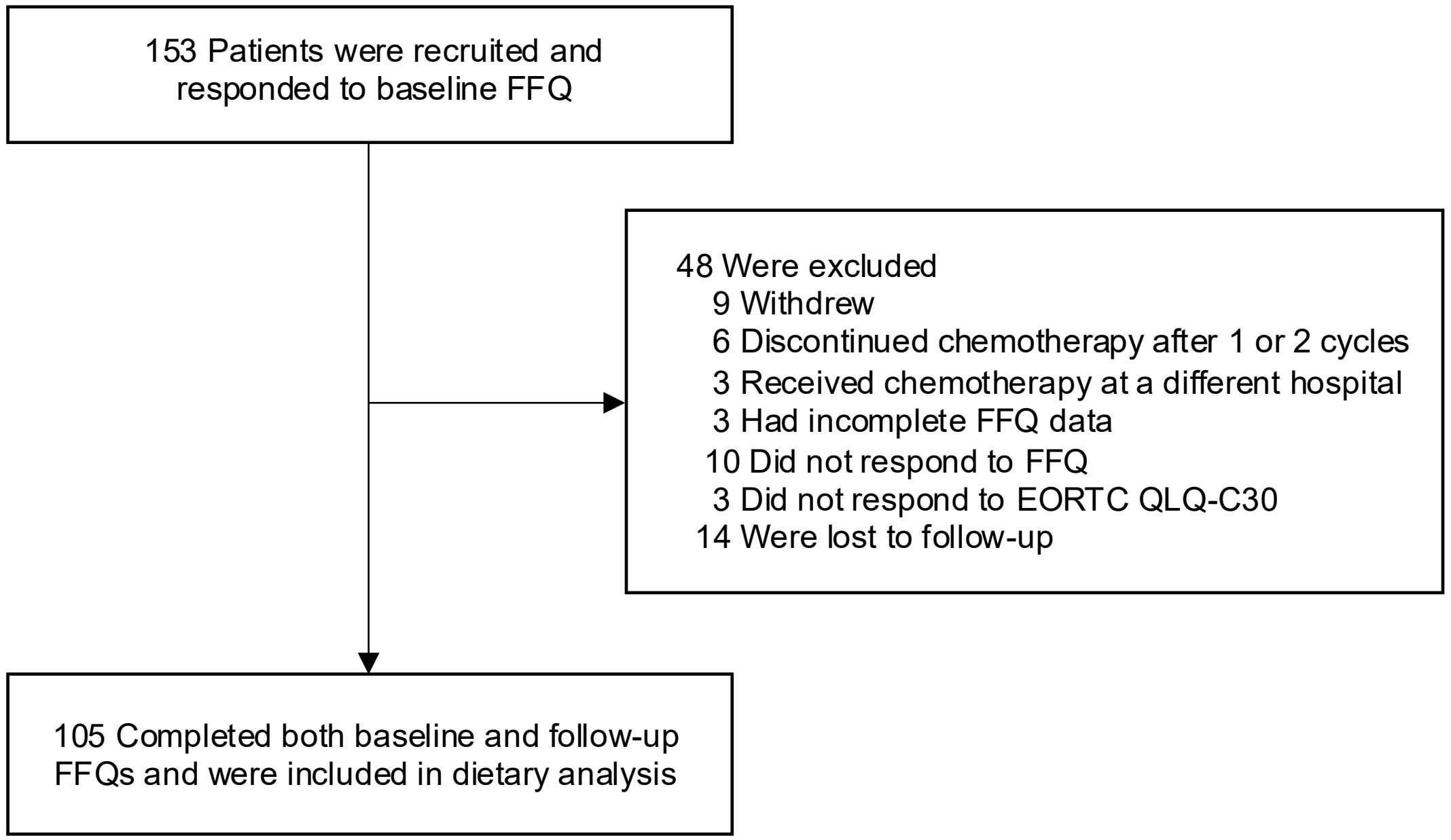

Figure S2. (a) Weekly changes in the Global Health Status (GHS)/QOL scores during chemotherapy. Boxes represent the interquartile range, with the horizontal line indicating the median. Black dots represent the mean score at each time point, and black lines connect the mean values across time points.

(b, c) SHAP summary (beeswarm) plot for the discrimination of (b) deterioration and (c) improvement in QOL based on changes in symptom scores. Feature ranking (y-axis) indicates the relative importance of each feature in the discrimination model. SHAP values (x-axis) indicate the contribution of each feature to model output. Each point represents an individual observation.

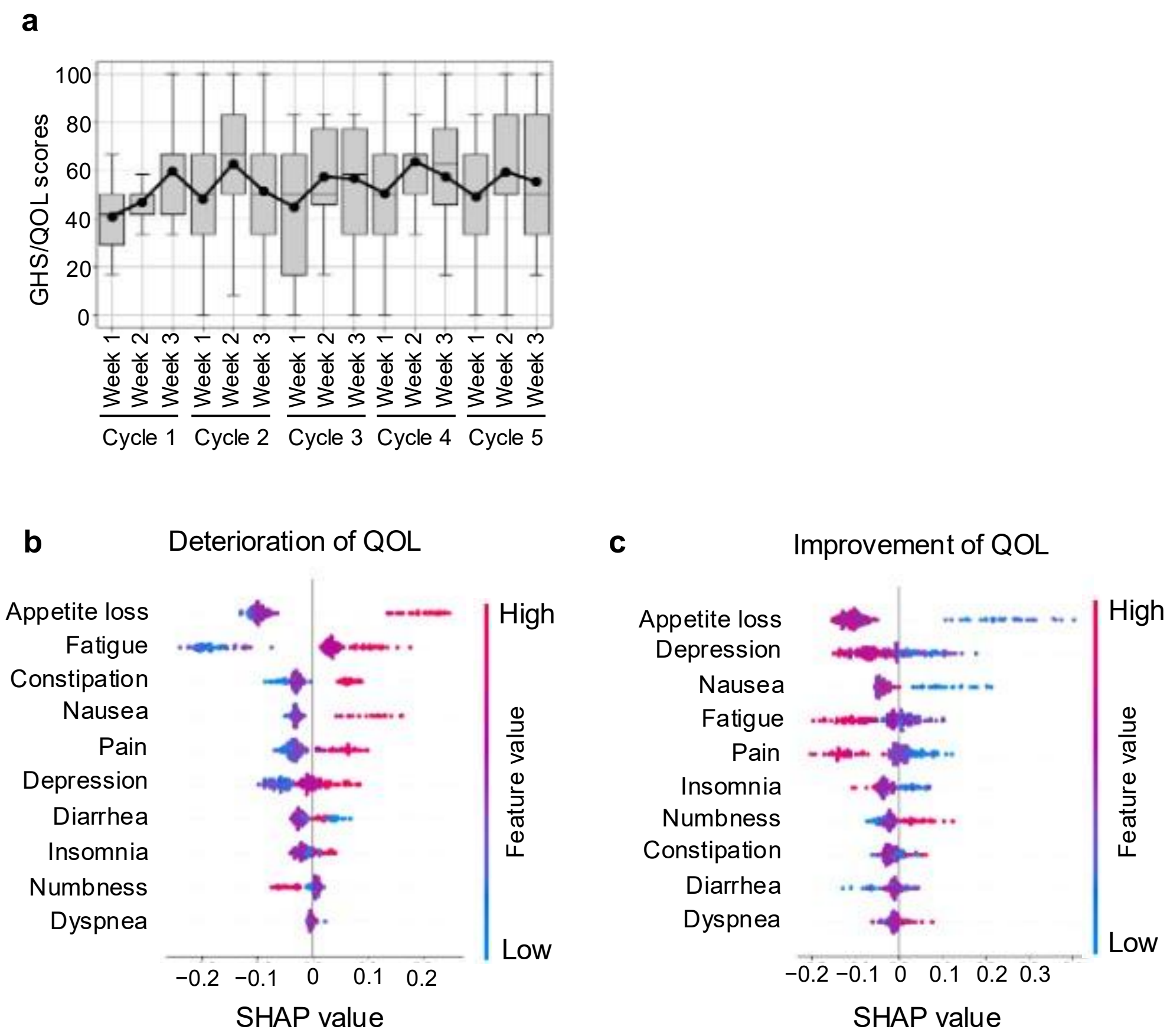

Figure S3. ROC curves for the test sets, illustrating the discriminative performance of RF, LightGBM, and XGBoost models for classifying (a) deterioration and (b) improvement of Global health status.

RF Random Forest, *LightGBM* Light Gradient Boosting, *XGB* eXtreme Gradient Boosting

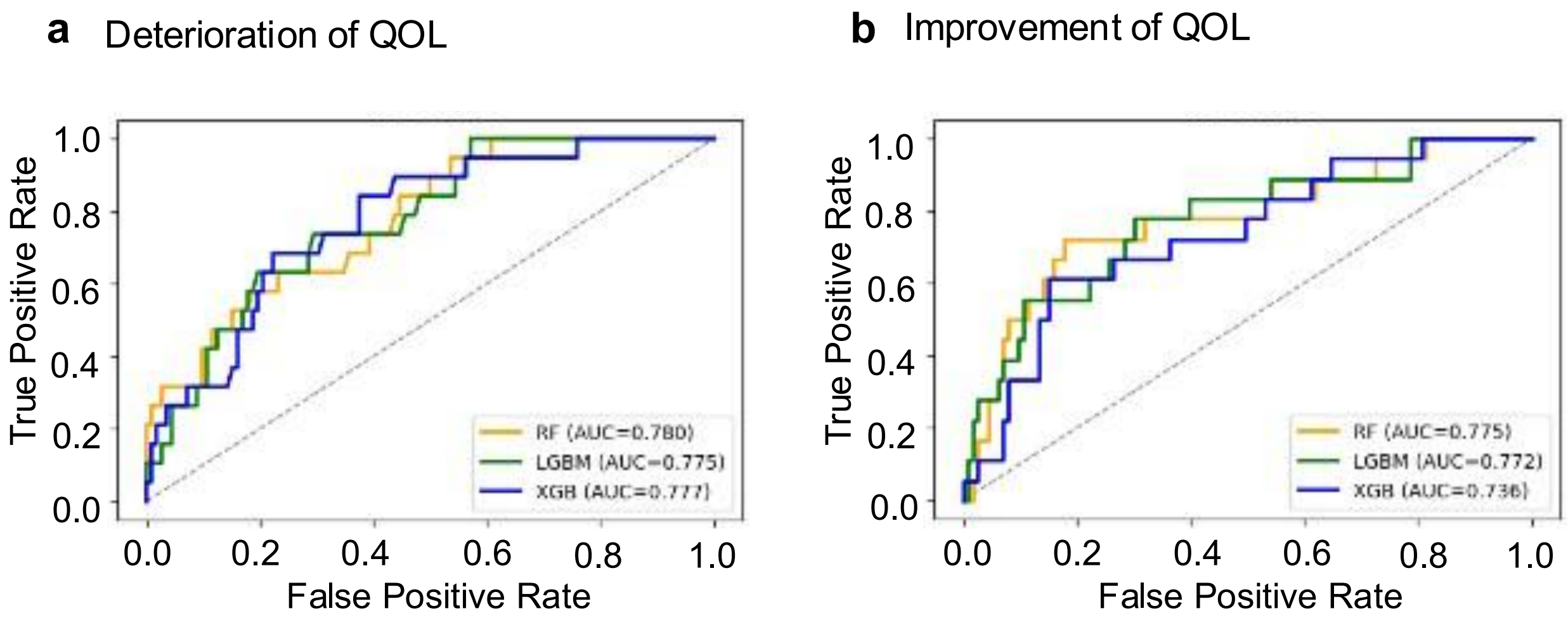

Table S1 Symptom scales data during chemotherapy

Scales	symptom	*n*	%
Fatigue	absent	9	8.6
	present	96	91.4
Nausea/vomiting	absent	73	69.5
	present	32	30.5
Pain	absent	35	33.3
	present	70	66.7
Dyspnea	absent	42	40.0
	present	63	60.0
Insomnia	absent	41	39.0
	present	64	61.0
Appetite loss	absent	64	61.0
	present	41	39.0
Constipation	absent	30	28.6
	present	75	71.4
Diarrhea [a]	absent	64	61.0
	present	40	38.1
Financial difficulties	absent	36	34.3
	present	69	65.7

[a] Data for diarrhea were missing for one patient

Table S2 Association between EORTC QLQ-C30 appetite loss scale and PRO-CTCAE decreased appetite severity at the third chemotherapy cycle

PRO-CTCAE decreased appetite severity	Appetite loss (*n* = 33) *n* (%)	No appetite loss (*n* = 48) *n* (%)
0	7 (21.2)	42 (87.5)
1	10 (30.3)	4 (8.3)
2	9 (27.3)	2 (4.2)
3	6 (18.2)	0 (0.0)
4	1 (3.0)	0 (0.0)

PRO-CTCAE decreased appetite severity data were available for 81 of 105 patients, because PRO-CTCAE assessment was introduced partway through the study period.

Table S3 Baseline, cycle 3, and changes in dietary intake by appetite loss status

Variable	Appetite loss			No appetite loss		
	Baseline median [IQR]	Cycle 3 median [IQR]	Change median [IQR]	Baseline median [IQR]	Cycle 3 median [IQR]	Change median [IQR]
Energy (kcal/day)	1588.4 [1053.7 to 1969.7]	1352.2 [1036.2 to 1672.0]	−226.1 [−382.1 to 62.0]	1473.8 [1189.8 to 1802.0]	1491.5 [1332.1 to 1832.4]	94.6 [−142.4 to 341.8]
Protein (g/day)	56.8 [39.3 to 85.6]	53.6 [38.6 to 71.4]	−7.1 [−15.1 to 1.4]	57.4 [46.9 to 76.2]	58.7 [51.3 to 84.2]	4.9 [−2.8 to 11.1]
Fat (g/day)	50.7 [32.8 to 61.8]	47.2 [32.1 to 53.6]	−6.3 [−16.6 to 1.9]	45.1 [36.0 to 58.2]	48.3 [37.1 to 60.0]	2.0 [−7.1 to 11.3]
Carbohydrate (g/day)	191.5 [141.9 to 263.1]	171.3 [138.9 to 229.3]	−9.9 [−67.6 to 24.1]	184.6 [145.1 to 231.2]	208.8 [175.7 to 249.5]	15.7 [−15.6 to 58.4]
Protein-to-energy ratio (%)	15.5 [14.2 to 18.3]	16.2 [14.7 to 17.5]	−0.2 [−1.5 to 1.6]	16.0 [14.9 to 18.9]	16.2 [14.7 to 18.7]	0.1 [−1.3 to 1.3]
Fat-to-energy ratio (%)	29.2 [25.5 to 34.1]	29.2 [24.7 to 32.9]	−1.5 [−3.1 to 2.7]	29.9 [25.3 to 33.6]	28.8 [25.2 to 30.8]	−1.3 [−4.2 to 2.8]
Carbohydrate-to-energy ratio (%)	51.5 [45.9 to 57.5]	53.8 [48.1 to 58.5]	0.3 [−2.3 to 6.9]	50.9 [45.7 to 56.6]	54.0 [49.2 to 57.0]	2.9 [−0.6 to 7.4]

Table S4 ANCOVA models for dietary intake at the third chemotherapy cycle according to appetite loss status

Outcome	Analysis	*n* [a]	Adjusted difference [b]	(95% CI)	*p* value
Energy (kcal/day)	Overall cohort [d]	100	−246.6	(−398.4 to −94.9)	0.002
	Postoperative cohort [c, d]	85	−182.5	(−351.4 to −13.6)	0.035
	+ adjustment for surgical factors [e]	85	−209.2	(−381.4 to −37.0)	0.018
Protein (g/day)	Overall cohort	100	−9.7	(−16.5 to −3.0)	0.005
	Postoperative cohort	85	−8.8	(−16.2 to −1.4)	0.020
	+ adjustment for surgical factors	85	−9.8	(−17.3 to −2.4)	0.010
Fat (g/day)	Overall cohort	100	−5.4	(−10.8 to −0.04)	0.048
	Postoperative cohort	85	−3.2	(−9.1 to 2.7)	0.288
	+ adjustment for surgical factors	85	−4.0	(−10.0 to 2.0)	0.188
Carbohydrate (g/day)	Overall cohort	100	−38.8	(−62.3 to −15.3)	0.001
	Postoperative cohort	85	−28.0	(−53.6 to −2.4)	0.033
	+ adjustment for surgical factors	85	−29.9	(−56.4 to −3.4)	0.028

[a] Five patients with missing baseline appetite loss scores were excluded from the ANCOVA analyses.

[b] Adjusted difference represents the estimated between-group differences in dietary intake at the third chemotherapy cycle between patients with and without appetite loss.

[c] The postoperative cohort included patients who received postoperative adjuvant chemotherapy.

[d] The overall cohort and postoperative cohort models were adjusted for the corresponding baseline dietary intake, baseline appetite loss score, and age.

[e] The adjustment for surgical factors models were additionally adjusted for surgical approach (open vs minimally invasive surgery) and the interval from surgery to chemotherapy initiation.

Table S5 Changes in dietary intake among patients without appetite loss at baseline (*n*=49)

Variable	Incident appetite loss [a] (*n*=16) Change median [b] [IQR]	No appetite loss (*n*=33) Change median [IQR]	*p* value
ΔEnergy (kcal/day)	−276.7 [−426.9 to −103.0]	45.0 [−282.5 to 239.6]	0.054
ΔProtein (g/day)	−9.2 [−12.5 to −0.6]	1.8 [−14.8 to 7.2]	0.14
ΔFat (g/day)	−8.6 [−15.0 to 2.5]	1.4 [−8.0 to 8.8]	0.24
ΔCarbohydrate (g/day)	−30.0 [−65.4 to 7.7]	1.8 [−20.7 to 31.9]	0.040
ΔProtein-to-energy ratio (%)	0.2 [−1.1 to 2.4]	−0.1 [−1.2 to 0.7]	0.45
ΔFat-to-energy ratio (%)	−0.7 [−2.3 to 3.0]	−0.9 [−4.2 to 3.4]	0.94
ΔCarbohydrate-to-energy ratio (%)	1.3 [−1.1 to 6.8]	3.3 [−0.3 to 7.7]	0.48

[a] The analysis was restricted to patients without appetite loss at baseline (EORTC QLQ-C30 appetite loss score = 0).

Incident appetite loss was defined as an appetite loss score > 0 at the third chemotherapy cycle.

[b] Changes were calculated as dietary intake at the third chemotherapy cycle minus the corresponding baseline dietary intake.

Between-group differences were assessed using the Mann–Whitney *U* test.

Table S6 Changes in dietary intake according to appetite loss severity categories

Variable	None (n=64) median [IQR]	A little (n=32) median [IQR]	Quite a bit/very much (n=9) median [IQR]	Spearman's ρ	p value
ΔEnergy (kcal/day)	94.6 [−142.4 to 341.8]	−170.1 [−307.0 to 34.9]	−303.6 [−406.7 to 113.7]	−0.33	< 0.001
ΔProtein (g/day)	4.9 [−2.8 to 11.1]	−6.2 [−16.3 to 2.6]	−10.6 [−13.5 to −0.8]	−0.34	< 0.001
ΔFat (g/day)	2.0 [−7.1 to 11.3]	−6.1 [−16.8 to 3.7]	−11.0 [−14.5 to 0.3]	−0.28	0.004
ΔCarbohydrate (g/day)	15.7 [−15.6 to 58.4]	−2.7 [−61.6 to 26.0]	−53.1 [−71.0 to −8.1]	−0.30	0.002
ΔProtein-to-energy ratio (%)	0.1 [−1.3 to 1.3]	−0.4 [−1.6 to 2.3]	−0.0 [−0.9 to 1.2]	−0.01	0.91
ΔFat-to-energy ratio (%)	−1.3 [−4.2 to 2.8]	−1.6 [−4.5 to 3.0]	−0.2 [−2.4 to 0.3]	0.01	0.92
ΔCarbohydrate-to-energy ratio (%)	2.9 [−0.6 to 7.4]	1.8 [−2.5 to 7.1]	−0.5 [−1.4 to 4.6]	−0.09	0.35

Table S7 Changes in micronutrient intake by appetite loss status during chemotherapy

Nutrient	Appetite loss		No appetite loss		Median difference	Hedges' *g*	*p* value	FDR-adjusted *p* value	Energy-adjusted *p* value [a]
	median	[IQR]	median	[IQR]					
Iron (mg)	−0.7	[−1.8 to 0]	0.9	[−0.5 to 1.7]	−1.5	−0.76	< 0.001	< 0.001	0.14
Zinc (mg)	−1.0	[−1.5 to 0.2]	0.6	[−0.5 to 1.5]	−1.7	−0.76	< 0.001	< 0.001	0.54
Magnesium (mg)	−23.1	[−54.5 to −1.4]	18.1	[−10.1 to 42.6]	−41.2	−0.72	< 0.001	< 0.001	0.36
Vitamin B_2 (mg)	−0.1	[−0.4 to 0.1]	0.06	[−0.1 to 0.3]	−0.2	−0.68	0.001	0.003	0.12
Potassium (mg)	−131.3	[−603.1 to 149.3]	223.5	[−113.7 to 531.5]	−354.8	−0.58	< 0.001	0.003	0.56
Vitamin B_6 (mg)	−0.1	[−0.2 to 0.06]	0.1	[−0.07 to 0.3]	−0.2	−0.57	< 0.001	0.002	0.59
Vitamin E (mg AT)	−0.6	[−1.5 to 0.2]	0.7	[−0.5 to 1.7]	−1.3	−0.56	0.001	0.003	0.77
Folate (µg)	−16.1	[−70.5 to 27.4]	37.4	[−11.9 to 82.5]	−53.5	−0.53	0.004	0.008	0.61
Vitamin B_1 (mg)	−0.04	[−0.2 to 0.03]	0.07	[−0.08 to 0.2]	−0.1	−0.52	0.007	0.013	0.76
Sodium (mg)	−277.1	[−704.0 to 510.1]	259.6	[−313.4 to 854.4]	−536.7	−0.50	0.017	0.023	0.18
Calcium (mg)	−27.1	[−132.3 to 49.8]	42.5	[−47.8 to 134.3]	−69.7	−0.50	0.014	0.021	0.53
Niacin (mg)	−1.4	[−4.2 to 0.7]	0.4	[−2.6 to 2.8]	−1.7	−0.48	0.014	0.021	0.89
Vitamin B_{12} (µg)	−0.7	[−2.6 to 1.1]	0.9	[−2.1 to 2.4]	−1.5	−0.47	0.034	0.041	0.73
Vitamin D (µg)	−0.9	[−4.5 to 2.7]	0.5	[−3.8 to 3.6]	−1.5	−0.39	0.19	0.19	0.73
Vitamin C (mg)	−0.9	[−25.4 to 21.5]	17.2	[−9.8 to 44.6]	−18.1	−0.31	0.049	0.056	0.87
Vitamin A (µg RAE)	−26.5	[−168.2 to 113.8]	39.7	[−139.6 to 235.3]	−66.2	−0.29	0.15	0.16	0.58

AT α-tocopherol, *RAE* retinol activity equivalents

[a] Energy-adjusted *p* values were calculated using changes in nutrient density (nutrient intake per 1000 kcal of energy intake)

Table S8 Nutritional outcomes and counseling by appetite loss status during chemotherapy

Variable	Appetite loss *n* (%) or mean (± SD)	No appetite loss *n* (%) or mean (± SD)	*p* value
Weight loss [a] > 5%			
Yes	11 (26.8)	14 (21.9)	0.56
No	20 (48.8)	38 (59.4)	
Missing information [b]	10 (24.4)	12 (18.8)	
ΔAlbumin (g/dL) [c]	0.03 (0.6)	0.03 (0.6)	0.97
ΔPNI	0.2 (5.8)	0.2 (5.5)	1.00
Nutritional counseling [d]	5 (12.2)	3 (4.7)	0.26

PNI Prognostic Nutritional Index (Onodera's PNI)

[a] Δ Weight loss was assessed by comparing body weight at the last available cycle of chemotherapy with baseline body weight

[b] Previous body weight was unavailable due to massive ascites, or body weight data during and after chemotherapy were missing

[c] Data for albumin were missing for six patients

[d] Nutritional counseling was defined as cancer-related dietary counseling provided between treatment initiation and the assessment before the third chemotherapy cycle

Table S9 Clinical backgrounds of study patients for complementary analysis

Characteristics	Training set (n = 80)<br>n (%) or mean (± SD)	Test set (n = 47)<br>n (%) or mean (± SD)	p value
Age (years)	51.4 (9.1)	53.9 (9.3)	0.13
Cancer type			
Ovary	37 (46.2)	22 (46.8)	0.39
Endometrium	21 (26.2)	17 (36.2)	
Cervix	20 (25.0)	8 (17.0)	
Others	2 (2.5)	0 (0)	
Cancer stage			
III/IV, n (%)	35 (43.8)	24 (51.1)	0.54
Cancer status			
Primary	56 (70.0)	41 (87.2)	0.046
Recurrence	24 (30.0)	6 (12.8)	
Chemotherapy regimen			
Taxanes ± platinum, n (%)	61 (76.2)	32 (68.1)	0.45
Others	13 (16.2)	12 (25.5)	
CCRT	6 (7.5)	3 (6.4)	

CCRT concurrent chemoradiotherapy

Table S10 Summary of QOL and symptom scales based on patient-reported outcomes

PRO	Variable	Mean (± SD)
EORTC QLQ- C30	Global health status	54.3 (19.8)
	Fatigue scale	43.1 (22.4)
	Insomnia scale	32.8 (29.9)
	Constipation scale	27.2 (20.4)
	Pain scale	26.3 (22.9)
	Dyspnea scale	24.3 (21.4)
	Appetite loss scale	20.8 (21.8)
	Diarrhea scale	16.6 (20.7)
	Nausea scale	9.3 (13.4)
PRO-CTCAE	Numbness	1.3 (1.0)
PHQ-9	PHQ-9 total score	5.3 (4.3)

PRO patient-reported outcome

Table S11 Predictive performance of models for QOL deterioration and improvement in the test set

(a) QOL deterioration

	AUC	Accuracy	F1 Score	Sensitivity	Specificity	PPV	NPV
RF	0.780	0.763	0.415	0.579	0.795	0.324	0.918
LightGBM	0.775	0.748	0.421	0.632	0.768	0.316	0.925
XGBoost	0.777	0.779	0.326	0.368	0.848	0.292	0.888

(b) QOL improvement

	AUC	Accuracy	F1 Score	Sensitivity	Specificity	PPV	NPV
RF	0.775	0.771	0.464	0.722	0.779	0.342	0.946
LightGBM	0.772	0.702	0.418	0.778	0.690	0.286	0.951
XGBoost	0.736	0.756	0.407	0.611	0.779	0.306	0.926

AUC Area under the curve, *PPV* Positive predictive value, *NPV* Negative predictive value, *RF* Random Forest, *LightGBM* Light Gradient Boosting, *XGBoost* eXtreme Gradient Boosting

References

1. Kroenke K, Spitzer RL, Williams JB (2001) The PHQ-9: validity of a brief depression severity measure. J Gen Intern Med 16(9):606-613. https://doi.org/10.1046/j.1525-1497.2001.016009606.x
